# Supplementary material for: Effect of Ginger on Chemical Composition, Physical and Sensory Characteristics of Chicken Soup
Source: Foods. 2021 Jun 23;10(7):1456. doi: 10.3390/foods10071456 (PMC8307344; doi:10.3390/foods10071456)
Supplement: Supplementary file 1 [file foods-10-01456-s001.zip › foods-1239806-SI.pdf]

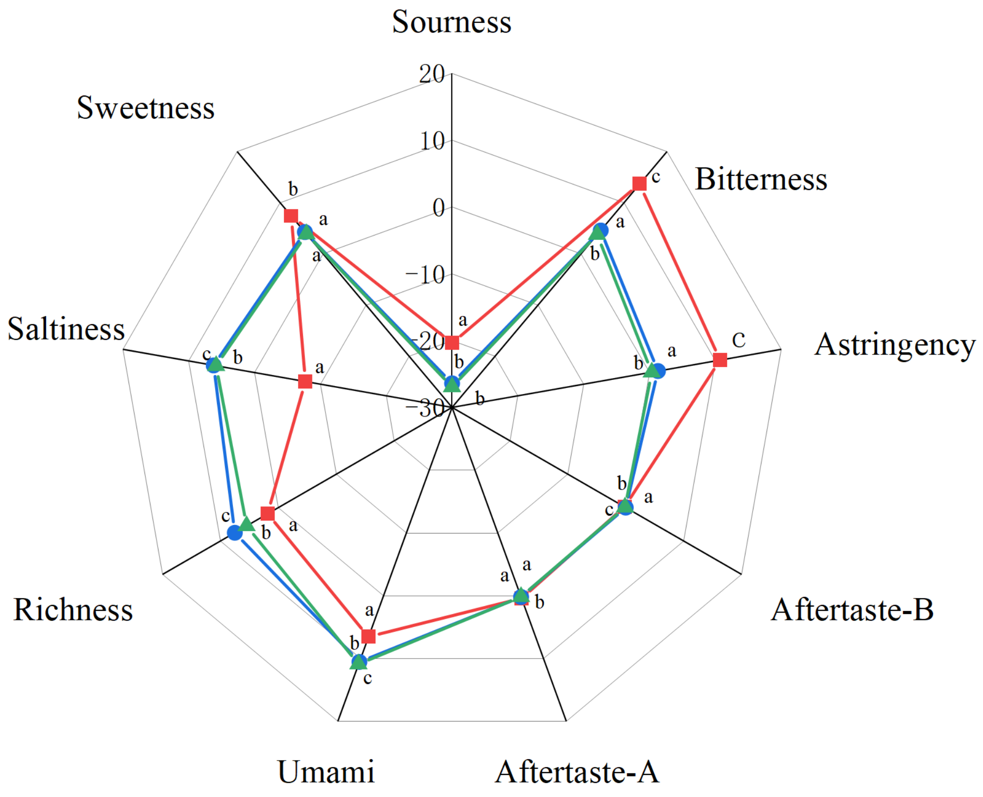

Figure S1. Radar chart of electronic tongue data with different soups. represents ginger soup, represents chicken soup, and represents ginger chicken soup.

Table S1. The color of different soups.

Samples	L *	A *	B *
Ginger soup	80.75 ± 0.10 [a]	−0.21 ± 0.04 [a]	3.22 ± 0.42 [a]
Chicken soup	85.03 ± 1.00 [b]	−0.20 ± 0.17 [a]	3.19 ± 1.47 [a]
Ginger chicken soup	79.69 ± 0.26 [a]	−0.04 ± 0.08 [a]	2.88 ± 0.74 [a]

Means in the same row with no common superscript differed significantly ($p < 0.05$).

Table S2. EUC values of different soups.

Sample	EUC（g MSG/100 g）
Ginger broth	0.06 ± 0.01 [a]
Chicken broth	16.64 ± 0.27 [b]
Ginger chicken broth	19.54 ± 1.35 [c]

Means in the same row with no common superscript differed significantly ($p < 0.05$).
